# Identifying Predictors of Early Growth Response and Adverse Radiation Effects of Vestibular Schwannomas to Radiosurgery

**DOI:** 10.1371/journal.pone.0110823

**Published:** 2014-10-22

**Authors:** Soroush Larjani, Eric Monsalves, Houman Pebdani, Boris Krischek, Fred Gentili, Michael Cusimano, Normand Laperriere, Caroline Hayhurst, Gelareh Zadeh

**Affiliations:** 1 Department of Neurosurgery, University Health Network, Toronto, Canada; 2 Department of Neurosurgery, St. Michael's Hospital, Toronto, Canada; 3 Radiation Oncology Program, Princess Margaret Hospital, Toronto, Canada; 4 Department of Neurosurgery, The Walton Centre, Liverpool, United Kingdom; University of Wisconsin School of Medicine and Public Health, United States of America

## Abstract

**Purpose:**

To determine whether pre-treatment growth rate of vestibular schwannomas (VS) predict response to radiosurgery.

**Methods:**

A retrospective review of a prospectively maintained database of all VS patients treated with 12Gy prescription dose between September 2005 and June 2011 at our institution using the Leksell Model 4C Gamma Knife Unit was conducted. Patients who had a minimum of 12-months clinical and radiological assessment before and after radiosurgery were included in this study. Tumor growth rates were calculated using specific growth rate (SGR). Tumor volumes were measured on FIESTA-MRI scans using ITK-SNAP v2.2.

**Results:**

Following radiosurgery, twenty-seven (42.9%) patients showed a significant decrease in volume after one year, twenty-nine (46.0%) stabilized, and seven (11.1%) continued to grow. There was no correlation between VS pre-treatment SGRs with post-treatment SGRs (*p* = 0.34), and incidence of adverse radiation effects (ARE). The reduction in tumors' SGRs after radiosurgery was proportional to pre-treatment SGRs, although this correlation was not statistically significant (*p* = 0.19). Analysis of risk factors revealed a positive correlation between post-treatment SGRs and incidence of non-auditory complications, most of which were attributed to ARE (*p* = 0.047).

**Conclusion:**

Pre-treatment growth rate of VS does not predict tumor response to radiosurgery or incidence of ARE. VS with higher SGRs post-radiosurgery are more likely to experience ARE.

## Introduction

Vestibular Schwanommas (VS) account for approximately 10% of all intracranial tumors [1], [2]. Stereotactic radiosurgery (SRS) is an accepted treatment option for VS that are less than approximately 3 centimeters in diameter. SRS effectively controls VS growth in more than 93% of cases [1], [2]. It also eliminates the high risk of irreversible cranial and facial nerve injury, and treatment-related morbidity that is associated with microsurgery. Nevertheless, SRS has associated ARE, such as radiation-induced cranial nerve and brain stem injury [3], [4]. In order to decrease the rate of adverse radiation effects (ARE), SRS prescription doses have steadily declined while attempting to maintain tumor control [1], [2]. Currently, the optimal recommended prescription dose is between 12–13Gy, where stable disease is achieved at a high rate, and the incidence of ARE is reduced to a minimum [1], [3]–[5].

One factor that has received little attention is whether the pre-treatment growth rate of VS can predict tumor response to radiosurgery, as well as the incidence of SRS complications or ARE. In other central nervous system tumor types, such as glioblastoma, pre-treatment tumor growth rate has been demonstrated to be a predictor of response to radiation therapy [6]. Majority of this research has focused on gliomas where biomathematical models have been developed that utilize a tumor's pre-treatment growth rate to predict response to radiation therapy [6]. In this study we focused on determining whether pre-treatment growth rate of VS can predict the early or short term response to SRS and/or serve as a predictor of ARE.

## Methods and Materials

This retrospective study was approved by the Research Ethics Board at University Health Network. This was a retrospective study where patients were fully de-identified/anonymized.

### Patients

We retrospectively reviewed a prospectively maintained database of all VS cases treated at our institution with SRS between December 2005 and June 2011. A total of 258 patients with VS were treated at our institution with Leksell Model 4C Gamma Knife (Elekta Instrument, Atlanta, GA). At our institution, radiosurgery is indicated when there is documented evidence of tumor growth within 12 months of the first magnetic resonance imaging (MRI) scan, or when there is a strong patient preference for intervention. All patients were treated with a prescription dose of 12Gy. Treatments were planned using stereotactic CT and stereotactic contrast-enhanced 1.5-mm section T1 and T2 MRI sequences. All patients had standard clinical and radiological assessment 12 months prior to radiosurgery, and 3 months, 6 months, and 12 months after radiosurgery, and then yearly thereafter.

Patients who did not have a minimum of two pre-treatment and/or two post-treatment MRIs that were at least 12 months apart were excluded; this criteria was required in order for us to calculate the radiological growth rates of tumors before and after radiosurgery. In addition, patients who did not have appropriate and/or complete clinical assessment pre- and post-radiosurgery were excluded. We also excluded patients with tumors that underwent pseudoprogression following treatment. Pseudoprogression is the transient increase in tumor volume followed by stability or regression that commonly occurs after radiosurgery [3]. In specific, we defined pseudoprogression as an increase of more than 20% in the first year after radiosurgery, with tumor stabilization or regression within 24 months.

### Volumetric Measurements

In this study we evaluated tumor size and growth rate using volumetric measurements. ITK-SNAP v2.2 (University of Pennsylvania, Philadelphia, PA), which is a validated and reliable software for medical image segmentation, was used to examine axial MRI scans, and perform volumetric measurements [7]. The semi-automatic segmentation function was used with 1.5-mm section FIESTA MRI images for volumetric measurements. Manual contouring was used for post-semi-automatic segmentation processing, and for MRI images where differentiation of tumor mass from surrounding tissues was difficult. Two independent trained research assistants were used to delineate the tumor edge, and define image voxels as tumor and non-tumorous, after which measurements of tumor volume were calculated. Subsequently the measurements were verified by a neuroradiologist blinded to the previous measurements who used the same software setup for volumetry. All image reviewers were blinded to the time point of imaging with respect to treatment.

### Tumor Growth Rate Measurement

In order to study the relationship between pre-treatment and post-treatment tumor growth rates, patients who had clinical and radiological assessment at least 12 months before and after SRS were selected. Typically, patients had only one radiological assessment prior to radiosurgery, and that was performed 12 months prior to the operation in accordance with the ‘wait and scan’ policy. Tumor volumes were measured using axial FIESTA MRI scans obtained on appropriate dates, and were recorded in cubic centimeters. Tumor growth rates before and after SRS were calculated using the specific growth rate (SGR) formula: SGR  =  ln(V_2_/V_1_)/(t_2_-t_1_)x100%, where V_2_ and V_1_ equaled treatment volume and tumor volume 12 months before treatment, respectively, with t_2_ and t_1_ equaling corresponding time points. When calculating post-treatment SGRs, V_2_ and V_1_ equaled tumor volume 12 months post-treatment and treatment volume, respectively, with corresponding time points t_2_ and t_1_. SGRs were expressed as percent change per annum [8]. Significant growth after radiosurgery was defined as any volumetric growth greater than 20% over a 12-month period [3], [4], [9]. Tumor regression was defined as an annual volumetric decline of 20%. Annual volumetric changes between −20% and +20% was defined as stable disease; volumetric changes within ±20% are insignificant volumetric progressions or involutions. In a previous study we identified patients that had pseudoprogression [3]. Therefore these patients were excluded from this study to prevent bias in our results.

### Radiation Parameters

The dosimetric parameters for SRS were collected and listed in Table 1.

**Table 1:** Analysis Variables and Univariate Analysis.

		Post-treatment SGR		Reduction in SGR after radiosurgery	
Radiation Characteristics	Median (range)	Regression coefficient	*p*	Regression coefficient	*p*
Treatment time (min)	35.20(11.55–79.48)	0.15	0.25	−0.02	0.91
Dose Rate (Gy/min)	2.47(1.80–3.22)	−0.25	0.056	0.18	0.18
CI RTOG^4^	1.31 (1.06–1.86)	−0.03	0.83	0.10	0.48
Gradient Index	2.81 (2.42–4.03)	0.18	0.17	−0.23	0.46
Homogeneity index	2.00 (1.85–2.50)	0.13	0.35	−0.06	0.65
Volume receiving 100% dose (%)	99.12 (96.40–99.88)	0.12	0.38	−0.04	0.79
Target volume receiving 95% dose(%)	99.78 (98.34–99.99)	0.13	0.36	−0.08	0.58
Min dose (Gy)	10.08 (7.00–16.08)	−0.21	0.13	0.15	0.28
Max dose (Gy)	24.00 (22.22–30.10)	−0.09	0.49	0.09	0.51
Mean dose (Gy)	17.12 (15.61–19.37)	−0.13	0.15	0.28	0.08
Volume of 12Gy isodose (cm3)	1.67 (0.14–11.09)	−0.05	0.71	−0.01	0.93
Prescription isodose (%)	50 (40–54)	0.11	0.44	−0.10	0.46
Number of Isocentres	11 (2–22)	−0.01	0.93	0.04	0.79

### Adverse Radiation Effects

The following clinical symptoms reported after radiosurgery were recorded as ARE: impaired balance, trigeminal neuropathy, facial nerve dysfunction, tinnitus, and hydrocephalus. For the purposes of this study we only collected reports of new symptoms and not ongoing symptoms that would have existed prior to SRS treatment. We were unable to include auditory complications due to incomplete auditory clinical follow-up in this cohort of patients.

### Statistical analysis

Linear regression was used to assess the relation of pre-treatment SGR with post-treatment SGR and the extent of reduction in tumor growth rate after radiosurgery. To investigate the relation between age on date of treatment and SGRs, patients were categorized into two groups based on a cut-off age, and tested for correlation; cut-off ages tested were 60, 65, and 70. Independent sample t-test was used for comparison between two independent groups. One-way ANOVA was used for multiple independent group comparisons. Univariate analysis was used to evaluate risk factors in predicting tumor response and post-treatment complications, and to study the relation between the following variables: pre-treatment SGR, post-treatment SGR, ARE, treatment tumor volume, age, sex, and radiation parameters. For all tests, *p* values ≤0.05 were considered significant. All analyses were performed with IBM SPSS version 20.0 (SPSS, Chicago, IL, USA).

## Results

### Patient characteristics

Of the 258 VS patients treated with SRS between December 2005 and June 2011, 84 had complete clinical and radiological follow-up for at least 12 months before and after treatment, and therefore qualified for inclusion in this study; this inclusion criteria was necessary to allow us to calculate the tumor growth rates before and after SRS. Of the 84 patients, six patients were further excluded for having bilateral VS with neurofibromatosis type II. Ten other patients were excluded because of having either MRI scans with either poor resolution or greater than 3 millimeters thickness slice scans, which prevented accurate volumetric measurements. Five other patients were excluded as their tumors underwent pseudoprogression following treatment. In sum, a total of 63 patients with unilateral sporadic VS were included in this retrospective study. Twenty-eight (44.4%) patients were males and thirty-five (55.6%) were females. The median radiological and clinical follow-up after radiosurgery was 32 months (range, 12–72 months). The median age at treatment date was 64 years (range, 26–83 years). Twenty-eight patients (44.4%) had left-sided VS. The median tumor volume treated was 1.54 cm^3^ (range, 0.14–10.84 cm^3^).

### Tumor Growth Rate Characteristics

The median VS growth rates in the 12-month period before and after SRS were 55.3% (range, 2.6–172.8%) and −16.1% (range, −141.4–56.6%), respectively. Prior to radiosurgery, fifty-three (84.1%) VS had a significant growth over a 12-month period; their median pre-treatment SGR was 65.9%, and the median post-treatment SGR was -15.8%. One year following radiosurgery, twenty-seven VS (42.9%) showed a decrease in volume, twenty-nine (46.0%) stabilized, and seven (11.1%) continued to grow. Of the tumors that continued to grow, no stabilization or regression was evident at 24 months and therefore the continued growth was not explained by pseudoprogression. Radiosurgery was concluded to have failed in suppressing tumor growth in these patients. The average growth rate at 12 months post-radiosurgery for these tumors was 43.97%. Salvage microsurgical intervention was offered based on presence of symptoms secondary to mass effect or cranial nerve deficits caused by the growing tumors. Only one of the seven (1.6%) patients required salvage microsurgical resection because of symptomatic tumor growth. The pre-treatment growth rates for tumors that continued to grow after radiosurgery were highly variable, and were comparable to those that did not show growth following radiosurgery (i.e. tumor stabilization).

### Predictive factors associated with pre- and post-treatment growth rate

No significant correlation was found between pre-treatment or post-treatment tumor growth rates with any of the following factors that we investigated: tumor volume, patients' gender, or age at time of treatment (Table 2). To further investigate the relation between age on date of treatment and SGRs, patients were categorized into two groups based on a cut-off age, and tested for correlation; cut-off ages tested were 60, 65, and 70. No significant correlation was found between age groups and pre-treatment and post-treatment SGRs (Table 3).

**Table 2:** Correlation between tumor volume, sex, and age, with pre-treatment and post-treatment SGRs.

	Pre-treatment tumor SGR		Post-treatment tumor SGR	
	Regression Coefficient	*p*	Regression Coefficient	*p*
Tumor volume	0.014	0.92	0.033	0.80
Sex	0.112	0.11	0.206	0.36
Age on date of treatment	0.055	0.67	0.006	0.96

**Table 3:** Independent samples t-test for pre-treatment and post-treatment SGRs, with SGRs classified into two groups based on cut-off age values.

Cut-off age	No. (%)	Pre-treatment SGR *p* value	Post-treatment SGR *p* value
<60	24 (38.1)	0.56	0.72
>60	39 (61.9)		
<65	34 (54.0)	0.83	0.99
>65	29 (46.0)		
<70	50 (79.4)	0.75	0.36
>70	13 (20.6)		

### Pre-treatment growth rate as predictor of treatment response

**Figure 1:**
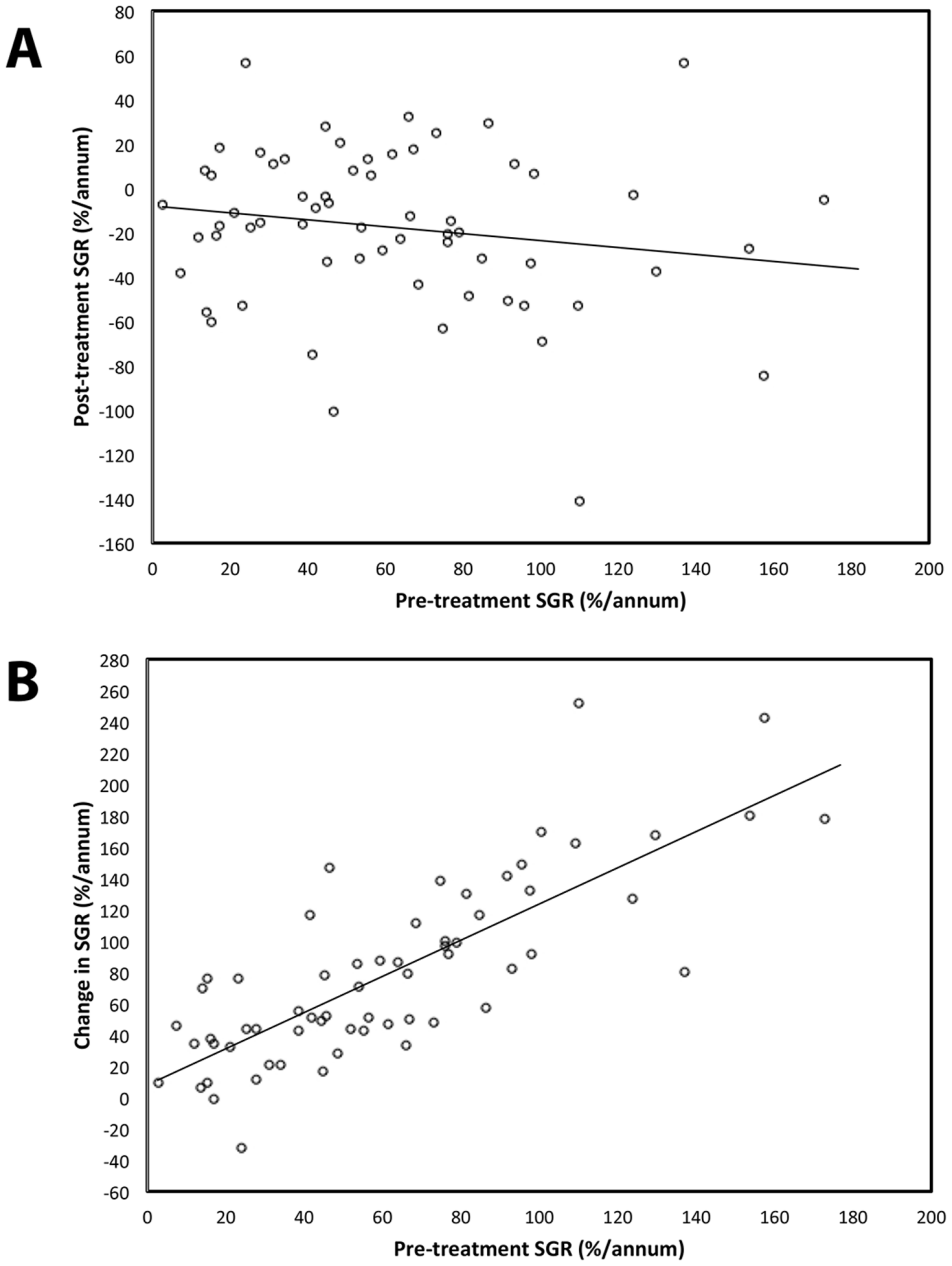
Correlation between pre-treatment SGRs with post-treatment SGRs, and reduction in SGRs after radiosurgery.

Linear regression between pre-treatment and post-treatment SGRs revealed no significant correlation (p = 0.34) (Figure 1a). However, we graphically observed that the growth rates of tumors reduced after radiosurgery in proportion to the tumors' initial pre-treatment growth rates (Figure 1b), although this relation was not statistically significant (R^2^ = 0.899, *p* = 0.19). We were then interested to see if VS can be categorized into clinically relevant subgroups based on their pre-treatment or post-treatment SGRs. We categorized tumors based on their response to SRS into three groups called regression, stable disease, and growth, using different cut-off values (Table 4), and attempted to see whether the pre-treatment growth rates between these groups significantly differ. We defined ‘regression’ as a decline in volume of more than the cut-off value, ‘growth’ as an increase in tumor volume of more than the cut-off value, and ‘stable disease’ as any volumetric change between ‘regression’ and ‘growth’. The cut-off values, although arbitrary, were chosen such that they could potentially be practically applied in a clincal setting. When we compared the the three groups, there was no significant difference between their pre-treatment growth rates (Table 4).

**Table 4:** a) Pre-treatment growth rates vs. post-treatment growth rate groups

Post-treatment growth rate	No. (%)	Median pre-treatment growth rate %/annum (range)	*p* value
10% cut-off value			
Regression	36 (57.1)	65.22 (7.37–157.60)	0.84
Control	12 (19.0)	45.00 (2.63–172.76)	
Growth	15 (23.9)	55.31 (17.10–136.87)	
15% cut-off value			
Regression	33 (52.4)	63.98 (7.37–157.60)	0.77
Control	19 (30.2)	45.45 (2.63–172.76)	
Growth	11 (17.4)	61.56 (17.10–136.87)	
20% cut-off value			
Regression	26 (41.3)	71.63 (7.37–157.60)	0.21
Control	30 (47.6)	45.00 (2.63–172.76)	
Growth	7 (11.1)	65.90 (24.16–136.87)	
33% cut-of value			
Regression	17 (27.0)	81.53 (7.37–157.60)	0.20
Control	44 (69.8)	52.56 (2.63–172.76)	
Growth	2 (3.2)	80.52 (24.16–136.87)	

b) Pre-treatment growth rates vs. post-treatment growth rate groups				
	Change in diameter (%/annum)	Change in volume (%/annum)	No. (%)	*p* value
First Test				
Group I	<10	<33	17 (27.0)	0.66
Group II	10–15	33–52	12 (19.0)	
Group III	>15	>52	34 (54.0)	
Second Test				
Group I	<10	<33	17 (27.0)	0.19
Group II	10–20	33–73	23 (36.5)	
Group III	>20	>73	23 (36.5)	
Third Test				
Group I	<15	<52	29 (46.0)	0.17
Group II	15–20	52–73	11 (17.5)	
Group III	>20	>73	23 (36.5)	

We also categorized tumors based on their pre-treatment growth rate into slow, medium, and fast growing tumors (called Group I, II, and III, respectively), and attempted to see whether the post-treatment growth rates significantly differ between these groups. We repeated the categorization using different cut-off values. The cut-off values were derived by converting changes in tumors' extrameatal diameter (10, 15, and 20%) that are commonly used in clinical practice, to volumetric changes by making the crude assumption that tumors are spherical; this conversion is commonly used in clinical practice. After categorizing the tumors into the aforementioned three groups, we compared their post-treatment SGRs; however, they did not significantly differ from each other (Table 4).

### Radiation Parameters

Univariate analysis of various dosimetry parameters examined (Table 1) showed no significant correlation with either post-treatment SGR or the extent of reduction in growth rate after radiosurgery.

### Adverse radiation effects

Of the 63 patients, twenty (31.7%) experienced some form of new onset complication after SRS that persisted in the time span of the study (Table 5). Of the seven patients whose tumor grew significantly after radiosurgery, only two (3.2%) experienced complications – one patient experienced facial weakness, while the other experienced facial spasms and imbalance. In these two cases, the complications may have been either due to tumor growth or ARE. In all other patients with tumors that did not exhibit significant growth, we attributed complications to ARE. The other 18 (28.5%) patients who experienced ARE had tumors that either stabilized or regressed after radiosurgery. Univariate analysis of risk factors revealed a significant positive relation between post-treatment SGRs and incidence of ARE (Table 5). Patients who experienced ARE had higher post-treatment growth rates than those who did not develop ARE. On multivariate analysis, no new factors were identified to be of significance.

**Table 5:** Univariate analysis for ARE.

ARE	No. (%)	Pre-treatment SGR	Post-treatment SGR	Age at treatment
		*p* value	*p* value	*p* value
Impaired balance	17 (27.0)	0.13	0.19	0.48
Tinnitus	8 (12.7)	0.74	0.23	0.62
Facial numbness	6 (9.5)	0.68	0.61	0.49
Facial palsy	3 (4.8)	0.48	0.59	0.64
Hydrocephalus	1 (1.6)	0.20	0.13	0.38
Adverse Event	20 (31.7)	0.63	0.047*	0.96

* Statistically significant.

## Discussion

SRS has proven to be a highly effective treatment for VS [1], [10], [11]. Nevertheless, a percentage of VS are resistant to radiosurgery, and continue to grow after treatment. There are currently no known factors that can predict which VS will fail to respond to radiosurgery. The traditional thinking has been that the rate of growth of a VS is a predictor of response to radiosurgery, and based on literature from other CNS tumors it is argued that a faster growing VS will respond more favorably to treatment [12]. We therefore sought to examine whether growth rate of VS prior to radiosurgery is a predictor of treatment response and alteration in tumor growth rate in a one year follow-up period following SRS.

The terminology used in the literature to define tumor size, growth, and control is highly variable, limiting our ability to compare results of radiosurgery between different institutions. Most institutions use a linear measurement for assessing VS size, which is then used to determine suitability for SRS and response to treatment. However linear measurements fail to accurately determine the actual tumor volume [10], [13], [14]. Volumetric measurements are by far a more reliable and accurate method for assessing three dimensional changes of tumor volume and tumor growth versus linear measurements. However, volumetric measurements are time consuming, which make their integration into clinical practice difficult.

There is currently no standardized system for reporting VS tumor growth rates [2], and as a consequence reports on natural history of VS are highly variable [10], [12], [15], [16]. Battaglia et al. conducted a retrospective study to assess the true effect of 12–13Gy radiation on VS by comparing the growth behavior of tumors with and without radiosurgery [15]. This group concluded that there is no significant difference between the growth patterns of untreated and radiosurgically treated VS particularly for small-sized tumors. However, Battaglia et al. used the Jackler staging system, which is a two-dimensional method, to determine tumor size, which does not measure the actual three-dimensional change in tumor volume.

In our study we used volumetric measurements to accurately quantify tumor growth. We also used a previously agreed upon definition of significant growth to define tumor control and tumor growth, which is any volumetric change greater than 20% as a significant change in tumor size [13]. Based on this definition the tumor control rate was 89% in the first year following SRS. We were able to demonstrate that regardless of tumor volume most VS respond favorably to 12–13Gy of radiation, and in following SRS have a steep decline in tumor growth rate. Furthermore, we found that the pre-treatment growth rates for tumors that continue to grow after radiosurgery are comparable to those that responded favorably to radiation and showed a decrease in growth. In other words there was no correlation between post-SRS continued growth and pre-treatment growth rate of VS. Tumors that continue growth after radiosurgery are most likely resistant to radiation because of varying intrinsic molecular properties [17]. It is important to acknowledge that in this study we examined the early changes in growth rates of VS following radiosurgery, and the results seen in early regression following treatment are not a unified concept and not necessarily directly correlated with long-term outcome. It is also important to note that increase in tumor volume following radiosurgery can be be due to either tumor cell proliferation or release of cytokines and inflammatory response following treatment, which the latter will not necessarily correlate to tumor progression or growth; however currently the two processes are indistinguishable clinically and radiologically.

We also observed a non-statistically singificant but compelling correlation between pre-treatment growth rate and change in growth rate after radiosurgery. We found that the change/reduction in the growth rates of most VS after radiosurgery was proportional to their initial pre-treatment growth rates; for instance, tumors with high growth rates before treatment had the greatest reduction in growth rate after radiosurgery, and tumors with initially low growth rates had the least reduction. The most obvious explanation for this finding is that in short-term, SRS halts tumor growth regardless of how fast the tumor is initially growing prior to radiosurgery, rather than causing cell death. Consequently, because fast growing tumors have high number of dividng cells, we see the greatest change in growth rate for tumors that have higher growth rates before radiosurgery. Furthermore, because SRS stops tumor growth irrespective of the initial growth rate, it suggests that at least in short-term, fast and slow growing tumors respond similarly to radiosurgery. These results are conflicting with the traditional thinking discussed earlier. It is therefore important for similar studies with larger sample sizes to be conducted to further explore and validate our findings on the response of fast and slow growing tumors to radiosurgery.

Furthermore, factors predicting the incidence of ARE are invaluable for clinical decision-making, and more importantly patient counseling. Numerous studies have focused on identifying such factors, and considerable attention has been given to treatment volume as a potential predictor [4], [5], [18], [19]. Tumors of large volume require a greater mass to be irradiated, which theoretically increases the amount of surrounding non-tumorous tissue exposed to radiation. Therefore it has been commonly hypothesized that VS with large volumes are more likely to be associated with ARE. In agreement with this theory, Friedman et al. found a significant correlation between treatment volume and incidence of ARE [19]. More recently, our group demonstrated that patients with VS larger than 5 cm^3^ are significantly more likely to develop ARE after radiosurgery [4]. Pre-treatment tumor growth has previously been speculated to be related to the incidence of ARE; Beegle et al. demonstrated that tumor growth before radiosurgery is a significant predictor of ARE. In this study we found no correlation between the two variables [18]. However, we did find that there is a significant correlation between tumors that continue to grow post-treatment and the incidence of non-auditory ARE. However, we are limited in this study by the small sample size and in particular with the number of patients that experienced complications following SRS. Therefore, ongoing multi-institutional studies with larger cohorts that are better powered and include longer patient follow-up are needed to more comprehensively study the relation between growth of VS and their response to radiosurgery, and incidence of ARE.

## Conclusion

Traditionally the rate of tumor growth prior to SRS has been considered to be a predictor of response to SRS and possibly ARE. In this study we found that the natural history of VS, most notably tumor growth, and target volume, do not serve as reliable predictors for response to radiosurgery in the early period following treatment. However, our systematic study of change in tumor growth rates demonstrates that VS growing at faster rates have greatest change in growth rate following radiosurgery. We also observed that patients with tumors that continue to grow at significant rates after radiosurgery are more likely to experience post-treatment ARE. Notably, VS that continue to grow following SRS have variable pre-treatment growth rates.
